# Mitochondrial genome sequencing of notorious scuticociliates (*Pseudocohnilembus persalinus*) isolated from Turbot (*Scophthalmus maximus* L.)

**DOI:** 10.1080/23802359.2018.1508388

**Published:** 2018-10-03

**Authors:** Yanqi Gao, Shibo Jin, Huifeng Dang, Shigen Ye, Ruijun Li

**Affiliations:** Agriculture and Rural Affairs Ministry of Key Laboratory of Mariculture & Stock Enhancement in North China's Sea, Dalian Key Laboratory of Marine Animal Disease Control and Prevention, College of Fisheries, Dalian Ocean University, Dalian, 116023, China

**Keywords:** Mitogenomics, *Pseudocohnilembus persalinus*, *Scophthalmus maximus* L, Scuticociliates

## Abstract

Previously, a pathogenic ciliate was isolated from the surface ulcer of a diseased Turbot (*Scophthalmus maximus* L.) at an aquaculture farm in North China. After morphological and molecular biological identification based on 18rRNA, the ciliated was identified as the notorious scuticociliates (*Pseudocohnilembus persalinus*). In this study, the whole sequence of the mitochondrial genomic gene of *P. persalinus* was carried out. The sequencing results showed that the complete sequence of *P. persalinus* mitogenome was 38,375 bp. There were 2 rRNAs, 4 tRNAs, and 34 protein-coding genes (PCGs), respectively, located on both the heavy strand and the light stand. 15 PCGs were on the heavy strand, and 19 PCGs on the light strand. Besides, phylogenetic trees among 11 ciliates were constructed based on the sequences of 17 PCGs located in mitogenome using BI methods. The results of clustering showed that *P. persalinus* and *Uronema marinum* was the first cluster belonging to the order Scuticociliatida. Our research results will further provide primary data for evolution and classified study of scuticociliates.

Turbot (*Scophthalmus maximus* L.) was a fast-growing, highly cold tolerance flatfish with high economic value, and had already become main factory-farmed species in North China (Lei et al. 2005). However, with the expansion of the size of Turbot farming, diseases followed, among which scuticociliatosis was especially serious in recent years (Cui et al. 2008). *Pseudocohnilembus persalinus* was very notorious as a representative scuticociliate (Kim et al, 2004).

In this study, the whole sequence of the mitochondrial genome of *P. persalinus,* previously isolated from the surface ulcer of a diseased Turbot (*S. maximus* L.) at an aquaculture farm in North China (38.9768 N, 121.3459 E), was sequenced and assembled by Illumina MiSeq Next-generation sequencing by SC Gene Company (Guangzhou, China). The sequence of *P. persalinus* mitochondrial genome was submitted and deposited in the GenBank (accession number MH608212). The results showed that the complete sequence of *P. persalinus* mitogenome was 38,375 bp. There were 2 rRNAs, 4 tRNAs (tRNA-Glu, tRNA-Phe, tRNA-His, and tRNA-Trp), and 34 protein-coding genes (PCGs), respectively, located on both the heavy strand and the light stand. 15 PCGs were on the heavy strand, and 19 PCGs on the light strand. Besides, 14 genes (*rpl14*, *ymf70*, *nad4*, *rps3*, *nad10*, *nad7*, *rps14*, *ymf75*, *atp9*, *nad3*, *cob*, *cox2*, *ymf56*, and *ymf67*) used the start codon ATG. Three genes (*cox1*, *rps13*, and *ymf64*) used the start codon ATA. Eight genes (*nad1_a*, *nad6*, *ymf66*, *rps19*, *nad2*, *ymf65*, *nad4l*, and *ymf68*) used the start codon TTA. Four genes (*rpl2*, *ymf63*, *yejR* and *nad5*) used the start codon ATT. Three genes (*rps12*, *rpl6*, and *rpl16*) used the start codon ATA. *nad1_b* used the start codon CTG. *nad9* used the start codon TAT. *ymf65* and *ymf64* used the stop codon TAG, and all other 32 PCGs used stop codon TAA.

Phylogenetic trees among 11 ciliates were constructed based on the sequences of 17 PCGs (*atp9, cox1, cox2, cytb, nad10, nad1_a, nad1_b, nad7, nd3, nd4, rpl14, rpl16, rpl2, rps12, rps13, rps14* and *rps19*) located in mitogenome using BI and ML methods. The results of clustering showed that *P. persalinus* and *Uronema marinum* was the first cluster belonging to the order Scuticociliatida (Figure 1). Moreover, *U. marinum* was also a representative scuticocilaite isolated from *Takifugu rubripes* previously (Li et al. 2018). The other ciliates were in obvious clades: Hymenostomatida, Peniculida, Urostylida, and Sporadotrichida (Figure 1).

**Figure 1.**
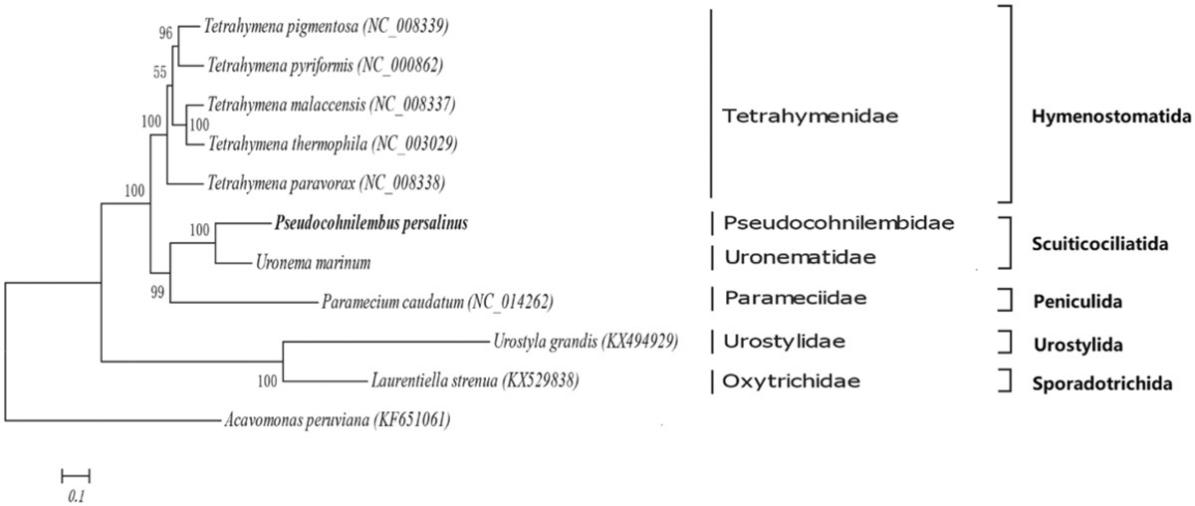
Phylogeny of *Pseudocohnilembus persalinus*. Phylogenetic tree based on the amino acid sequences of PCGs located in the mitogenome. The number of the branches denoted BI posterior probabilities.

## References

[CIT0001] Cui LB, Zhou XY, Cheng JW, Li YS, Ma JH 2008 Pathology of scuticociliatosis in *Scophthalmus maximus L*. J Fish Sci China. 15:622–629.

[CIT0002] Kim SM, Cho JB, Lee EH, Kwon SR, Kim SK, Nam YK, Kim KH 2004 *Pseudocohnilembus persalinus* (Ciliophora: Scuticociitida) is an additional species causing scuticociliatosis in olive flounder *Paralichthys olivaceus*. Dis Aquat Org. 62:239. 10.3354/dao062239 15672880

[CIT0003] Lei JL, Ma AJ, Chen C, Zhuang ZM 2005 The present status and sustainable development of turbot (*Scophthalmus maximus L.*) culture in China. Eng Sci. 7:30–34.

[CIT0004] Li RJ, Gao YQ, Hou YL, Ye SG, Wang LS, Sun JX, Li Q 2018 Mitochondrial genome sequencing and analysis of scuticociliates (*Uronema marinum*) isolated from *Takifugu rubripes*. Mitochondrial DNA Part B. 3:736–737. 10.1080/23802359.2018.1483757 PMC7800822 33474304

